# Supplementary material for: Lipidomic Analysis to Assess the Correlation between Ceramides, Stress Hyperglycemia, and HbA1c in Acute Myocardial Infarction
Source: Molecules. 2023 Jan 11;28(2):716. doi: 10.3390/molecules28020716 (PMC9862855; doi:10.3390/molecules28020716)
Supplement: Supplementary file 1 [file molecules-28-00716-s001.zip › molecules-2129769-supplementary.pdf]

SUPPLEMENTARY DATA

Chemicals and materials

Ammonium formate (14266), eluent additive for liquid chromatography-mass spectrometry (LC-MS), was purchased from Fluka Analytical (Sigma-Aldrich, St. Louis, MO, USA). LiChrosolv methanol (106035) and propan-2-ol (102781), both hypergrade solvent for LC-MS, were purchased from Supelco (Merck KGaA, Darmstadt, Germany) while chloroform (366927, for HPLC ≥ 99.8%) and DMSO (276855) from Sigma-Aldrich (St. Louis, MO, USA). Milli-Q deionized water was filtered on Millipak filter (0.22 μm, MPGL040001) and purified on a LC-Pak cartridge (C18, LCPAK0001) (all Millipore, Bedford, MA 01730, USA). Ceramide calibration standard (Cer(d18:1/17:0), 860517P) and N,N-dimethylsphingosine (d18:1) (DMS, 860496O) selected as internal standard (ISTD), were both purchased from Avanti Polar Lipids (Alabaster, USA).

Standard stock solutions

The stock solutions of Cer(d18:1/17:0) in $CHCl_3$/MeOH 70/30 and N,N-dimethylsphingosine in DMSO (respectively 1.5 mM and 15.3 mM) were prepared, stored and manipulated as previously described in E. Michelucci et *al*.[1].

Cer(d18:1/17:0) calibration curve preparation

First, ISTD stock solution was diluted with MeOH to the final concentration of 0.057 μM. Then, nineteen calibration levels, ranging from 222.2 to 0.00085 μM, were prepared for Cer(d18:1/17:0) according to the following procedure. A volume of 29.6 μl of calibration STD stock solution was added to 160.4 μl of MeOH (final volume 190 μl). This solution was split in two aliquots of 95 μl each. Five μl of 0.057 μM DMS were added to the first aliquot, thus creating the first calibration level (222.2 μM calibration STD, 0.00285 μM ISTD), while the second one was two-fold diluted with MeOH. The latter solution was in turn split in two aliquots of 95 μL each and sequential two-fold

dilutions were carried out till the 19th calibration level (0.00085 μM calibration STD, 0.00285 μM ISTD). Finally, the specific range 0.00085-0.434 μM of calibrators was selected in order to build the external calibration curve for Cer(d18:1/17:0).

Table S1. Table used for the MRM analysis of the 2 STDs and the SRM analysis of 15 Cer lipid species (dwell time 20 msec). The *m/z* values are calculated using the monoisotopic molecular weights. Q1 = first quadrupole of the mass spectrometer for precursor ion selection. Q3 = third quadrupole of the mass spectrometer for product ion selection. For the STDs are reported the optimized CEns to generate their quantifier (a) and qualifier (b) transitions. The transitions selected for the 15 Cer lipid species were based on the quantifier ion of the corresponding external standard. R = alkyl chain of the amide group in Cer.

**Lipid species**		**Precursor ion type**	**Precursor ion *m/z* (Q1)**	**CEn (V)**	**Product ion *m/z* (Q3)**	**Product ion type**
STD	Cer(d18:1/17:0)[a]	$[M+H]^+$	552.5	29	264.3	$Precursor^+$ -RCOOH $-H_2O$
STD	Cer(d18:1/17:0)[b]	$[M+H]^+$	552.5	29	282.3	$Precursor^+$ -RCOOH
STD	ISTD DMS(d18:1)[a]	$[M+H]^+$	328.3	26	310.3	$Precursor^+$ $-H_2O$
STD	ISTD DMS(d18:1)[b]	$[M+H]^+$	328.3	26	280.3	$Precursor^+$ $-CH_2O$ $-H_2O$
1	Cer(d18:0/16:0)	$[M+H]^+$	540.5	29	266.3	$Precursor^+$ -RCOOH $-H_2O$
2	Cer(d18:0/18:0)	$[M+H]^+$	568.6	29	266.3	$Precursor^+$ -RCOOH $-H_2O$
3	Cer(d18:0/22:0)	$[M+H]^+$	624.6	29	266.3	$Precursor^+$ -RCOOH $-H_2O$
4	Cer(d18:0/24:0)	$[M+H]^+$	652.7	29	266.3	$Precursor^+$ -RCOOH $-H_2O$
5	Cer(d18:0/24:1)	$[M+H]^+$	650.6	29	266.3	$Precursor^+$ -RCOOH $-H_2O$
6	Cer(d18:1/16:0)	$[M+H]^+$	538.5	29	264.3	$Precursor^+$ -RCOOH $-H_2O$
7	Cer(d18:1/18:0)	$[M+H]^+$	566.6	29	264.3	$Precursor^+$ -RCOOH $-H_2O$
8	Cer(d18:1/20:0)	$[M+H]^+$	594.6	29	264.3	$Precursor^+$ -RCOOH $-H_2O$
9	Cer(d18:1/22:0)	$[M+H]^+$	622.6	29	264.3	$Precursor^+$ -RCOOH $-H_2O$
10	Cer(d18:1/23:0)	$[M+H]^+$	636.6	29	264.3	$Precursor^+$ -RCOOH $-H_2O$
11	Cer(d18:1/24:0)	$[M+H]^+$	650.6	29	264.3	$Precursor^+$ -RCOOH $-H_2O$
12	Cer(d18:1/24:1)	$[M+H]^+$	648.6	29	264.3	$Precursor^+$ -RCOOH $-H_2O$
13	Cer(d18:1/25:0)	$[M+H]^+$	664.7	29	264.3	$Precursor^+$ -RCOOH $-H_2O$
14	Cer(d18:2/22:0)	$[M+H]^+$	620.6	29	262.3	$Precursor^+$ -RCOOH $-H_2O$
15	Cer(d18:2/23:1)	$[M+H]^+$	632.6	29	262.3	$Precursor^+$ -RCOOH $-H_2O$

REFERENCES

1. Michelucci E., Di Giorgi N., Finamore F., Smit J.M., Scholte A.J.H.A., Signore G., Rocchiccioli S. Lipid biomarkers in statin users with coronary artery disease annotated by coronary computed tomography angiography. *Sci. Rep.* 11, 12899 (2021). https://doi.org/10.1038/s41598-021-92339-0
